# Comprehensive Sensory Evaluation in Low‐Fat Emulsions: A Systematic Review of Diverse Food Applications

**DOI:** 10.1002/fsn3.4700

**Published:** 2024-12-20

**Authors:** Clara Talens, Saioa Alvarez‐Sabatel, Esther Sanmartín, Laura Garcia‐Fontanals, Pau Talens

**Affiliations:** ^1^ AZTI, Food Research, Basque Research and Technology Alliance (BRTA) Parque Tecnológico de Bizkaia, Astondo Bidea Derio Bizkaia Spain; ^2^ Instituto Universitario de Ingeniería de Alimentos—FoodUPV Universitat Politècnica de València Valencia Spain

**Keywords:** consumer acceptance, emulsifying technologies, fat replacers, low‐fat emulsions, sensory analysis

## Abstract

The prevalence of diet‐related health issues has driven the demand for healthier food options, particularly those with reduced fat content. This systematic review evaluates the integration of sensory analysis in low‐fat emulsion research, highlighting a significant gap in current practices. From an initial pool of 400 articles, 227 unique studies were screened, but only 15 (6.6%) included sensory analysis, underscoring a major shortfall in evaluating consumer acceptance. The reviewed studies investigated various emulsion types, including simple emulsions, emulsion gels, and Pickering emulsions, utilizing a diverse range of fat replacers, such as plant‐based oils, proteins, and modified starches. These fat replacers included natural and modified ingredients such as banana peel flour, lard‐based diacylglycerols, cedar oil cake, microparticulated egg white proteins, *Nigella sativa* oil, avocado, whey protein, flaxseed oil, polyphenol extracts, okara, microcrystalline wax and cellulose, rapeseed cake, and polysaccharide nanoparticles. These innovative approaches aimed to improve the sensory attributes of meat products, dairy‐type applications, salad dressings, and bakery products. The review highlights a disparity in the rigor and comprehensiveness of sensory evaluations among studies. While some studies have thoroughly assessed multiple attributes, others have been limited to general acceptability. This variability underscores the need for standardized, detailed sensory analysis in low‐fat emulsion research to ensure a comprehensive understanding of consumer preferences and product quality.

## Graphical abstract

This systematic review investigates the sensory and instrumental techniques used to evaluate low‐fat emulsions across various food applications. The study highlights the gaps in sensory analysis and suggests improved methodologies for assessing the quality and consumer acceptability of low‐fat emulsion products.

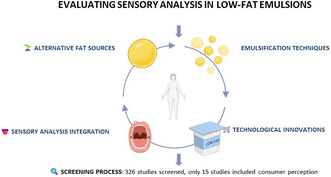

**Abbreviations**
CSD: Commercial salad dressing
DAG: Diacylglycerols
EG: Emulsion gel
FPOE: Flammulina velutipes polysaccharide nanoparticle‐palm oil emulsion
GL: Glycerolized lard
ME: Mint extract
MEWP: Microparticulate egg white protein
MUFAs: Monounsaturated fatty acids
MWP: Microparticulate whey protein
NDC: Non‐Dairy Creamer
O: Okara
PGL: Purified glycerol‐treated lard
PUFAs: Polyunsaturated fatty acids
PV: Peroxide value
QDA: Quantitative descriptive analysis
RF: Reduced fat
RSM: Response surface methodology
SF: Standard fat
SFA: Saturated fatty acids
SFC: Solid fat content
SO: Sunflower oil
TDS: Temporal dominance of sensations
WP: Whey protein

## 1 Introduction

Fat perception in food is a complex sensory experience that significantly influences consumer preferences. The relationship between fat content and sensory attributes is fundamental to the acceptance of various foods (Nieto and Lorenzo 2021). Studies by Frank‐Podlech et al. (2019) revealed that fat is associated with creaminess, fattiness, and palatability, which enhance the eating experience.

Recently, there has been a demand for healthier food options, with a focus on reducing fat content. The consumption of dietary fat has been shown to impact health outcomes, including obesity and cancer progression, highlighting the complex relationship among fat types, metabolic health, and disease mechanisms (Liu et al. 2020). This shift has led the food industry to explore innovative strategies for creating low‐fat alternatives without compromising the sensory pleasure (Liu et al. 2015; Nieto and Lorenzo 2021; Shi et al. 2024). The development of low‐fat food products poses significant challenges, including maintaining texture, flavor, and consumer satisfaction. Chen, Qin, and Zhang (2024) provide a comprehensive overview of challenges in fat reduction strategies and the role of flavors in addressing these issues. Achieving this balance requires understanding the mechanisms through which fat perception is influenced by the physicochemical properties of food, particularly in emulsion‐based products. Recent studies have explored plant‐based double emulsions as promising strategies in low‐fat food formulations, demonstrating their potential to enhance nutritional quality and sensory attributes (Huang et al. 2024).

The food industry faces a significant challenge: Reducing fat content while maintaining sensory qualities. Sensory analysis has become a critical tool in the development of low‐fat food products, providing insights into consumer perceptions that guide product formulation. Advancements in fat replacer design, including innovative sensory evaluation methods, have further expanded the understanding of consumer preferences for low‐fat products (Gao et al. 2024). Despite this, there is a lack of comprehensive sensory evaluations in studies on low‐fat emulsions, leading to a gap between product development and consumer satisfaction.

Food emulsions are colloidal systems used in the food industry to combine immiscible phases, typically oil and water, stabilized by emulsifiers (Lett, Norton, and Yeomans 2016, Liu et al. 2018). The physicochemical properties of these emulsions play a central role in shaping fat perception. The structural nature of emulsions allows for the controlled release of fat molecules during oral processing, influencing mouthfeel, flavor release, and overall satisfaction (Liu et al. 2015; Wu et al. 2023).

The classification of emulsions includes various dimensions, such as the type of phase (oil‐in‐water or water‐in‐oil), droplet size (macroemulsion, microemulsion, or nanoemulsion), and complexity of their structure (simple, multiple emulsions, or emulsion gels). The physicochemical properties of food emulsions are central to fat perception. Interfacial properties, particle size distributions, and rheological properties influence sensory attributes such as creaminess and richness. Understanding these interactions provides a scientific basis for designing processed foods that deliver desirable sensory experiences while meeting consumer demand for healthier options.

Emulsifiers control interfacial properties, significantly influencing fat distribution during oral processing. The choice of emulsifier affects the interfacial layer, impacting fat release and sensory perception (Gomes, Costa, and Cunha 2018; Hildebrandt et al. 2018; Zhang et al. 2021; Zhu et al. 2018).

The particle size distribution in oil‐in‐water emulsions affects fat perception. Smaller droplets enhance the perception of fat by increasing interactions with oral surfaces. Fine droplets contribute to a smoother texture, which is essential for mimicking full‐fat products (Zhou et al. 2019).

The rheological properties of emulsions, including viscosity and shear‐thinning behavior, significantly impact fat perception during consumption. Higher viscosity enhances mouth‐coating sensation, whereas shear‐thinning behavior contributes to a smoother mouthfeel (Klojdova and Stathopoulos 2022; Tenorio‐Garcia et al. 2022; Zhi et al. 2022).

Processing affects emulsions' structural characteristics, droplet size distribution, and interfacial properties, influencing their stability, texture, and fat perception. Understanding this evolution is imperative for developing emulsion‐based products that increase quality and sensory satisfaction.

The objective of this systematic review is to identify the most effective formulation and processing techniques for developing low‐fat emulsions across various food applications. The efficacy of these techniques was evaluated by examining their effects on the physicochemical properties and sensory characteristics of meat products, dairy‐type products, salad dressings, and bakery products. It focuses on studies bridging the gap between techno‐functional properties and sensory analysis, underscoring the importance of integrating sensory analysis with physicochemical evaluations to develop low‐fat emulsions that meet consumer expectations.

## 2 Methodology

### 2.1 Information Sources and Search Strategy

For this review, the Preferred Reporting Items for Systematic Reviews and Meta‐Analyses (PRISMA) 2020 statement was used (Page et al. 2021). The search strategy employed for this review aimed to comprehensively capture relevant literature pertaining to the influence of food processing on emulsion‐based fat perception. A systematic search was conducted across Web of Science and Scopus covering the period from January 2018 to September 2024. These databases were chosen because of their wide coverage of peer‐reviewed articles within the fields of food science, emulsion, and sensory perception.

The search strategy used was “emulsions and food processing and low fat” from 2018 to 2024.

The term “low fat” was employed as the primary search criterion to identify studies relevant to the physicochemical, nutritional, and sensory properties of low‐fat emulsions. Importantly, the use of “low fat” in our search strategy was intended to broadly encompass studies that investigate formulations with reduced fat content rather than those that strictly adhere to regulatory definitions. Regulatory guidelines specify that “low‐fat” foods must contain no more than 3 g of fat per serving. However, the scope of our review encompasses a wider range of fat reductions in emulsions, which may exceed this threshold. This approach included a broader spectrum of research findings relevant to fat reduction techniques and their impacts, regardless of strict legislative compliance. Thus, while some studies included in the review might refer to formulations that technically qualify under the “reduced fat” classification owing to their higher fat content, they are integral to understanding the overarching impact of fat reduction in food emulsions. We acknowledge that this may differ from legislative labeling requirements; however, the inclusion criteria were designed to capture the most comprehensive body of research applicable to the topic without being constrained by varying global definitions of fat content labelling.

### 2.2 Eligibility Criteria

The following articles were included: (1) focused on emulsifying technologies—specifically targeted studies involving the creation, formulation, or modification of emulsions for use in food products; (2) addressed low‐fat product development—articles had to discuss strategies, techniques, or outcomes related to reducing the fat content in food products through the use of emulsions; (3) included sensory analysis—required the inclusion of sensory evaluation methods to assess the taste, texture, aroma, or overall acceptability of the low‐fat products among consumers; (4) were original research; (5) were published in English; and (6) were peer‐reviewed publications. Review articles, opinion pieces, and editorials were excluded because they focused on empirical evidence.

### 2.3 Screening and Data Extraction

These articles were further screened via Covidence systematic review software (Veritas Health Innovation, Melbourne, Australia) on the basis of titles, abstracts, and relevance to the scope of the review.

### 2.4 Qualitative Synthesis

A detailed analysis of various physicochemical, nutritional, and sensory properties of low‐fat emulsions was performed across the included studies. The emulsion type, aim of the study, methodology, ingredients used, food application, textural parameters, viscosity, and sensory attributes were considered for each study. All the information was collected as a narrative review and is also available in the Supporting Information. The main findings and the quality of the sensory analysis were assessed.

## 3 Results and Discussion

### 3.1 PRISMA Flow Diagram and Study Selection

**FIGURE 1:**
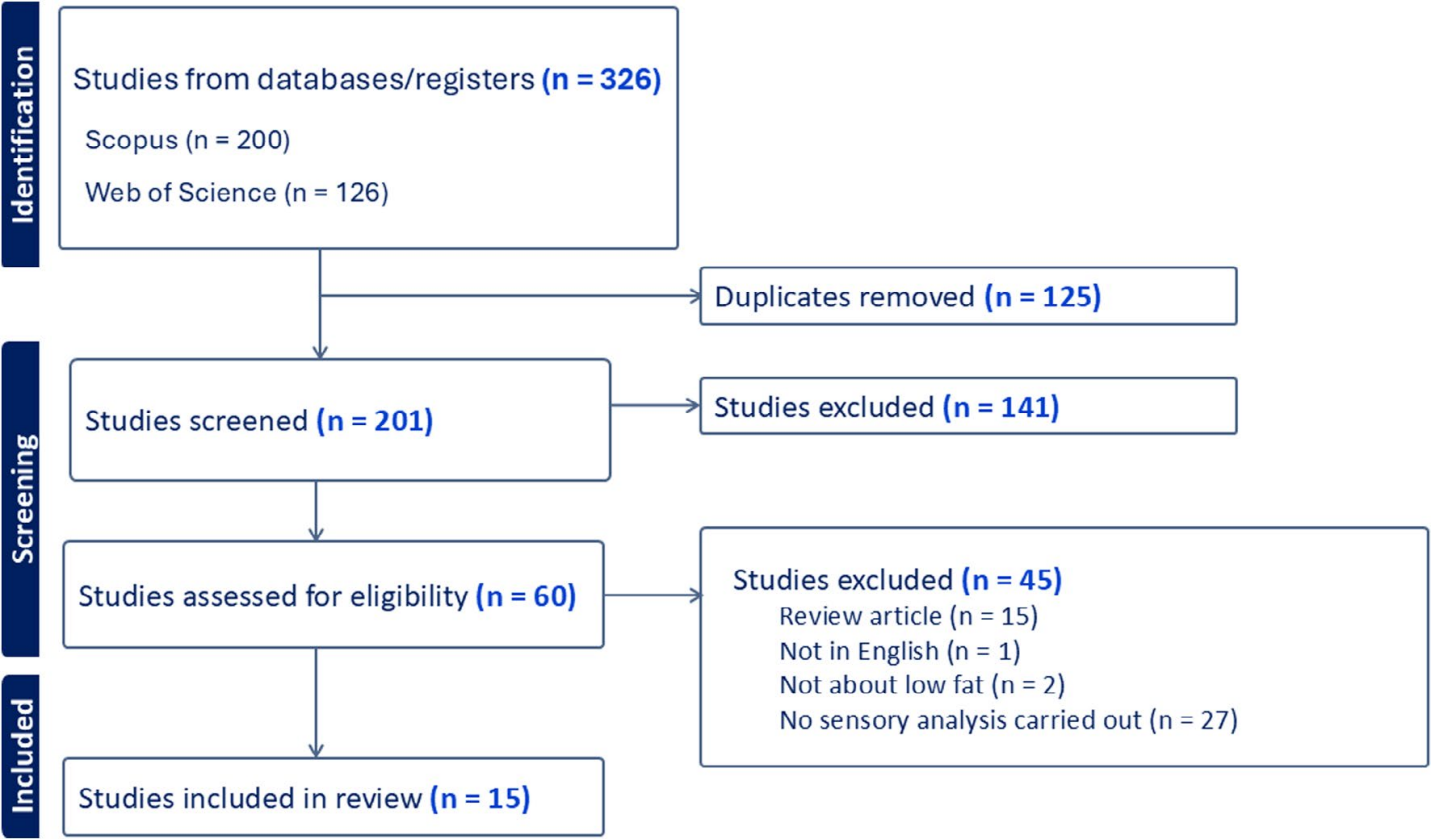
Flow diagram of the article search and selection process for the systematic review.

Figure 1 shows the PRISMA flow diagram outlining the identification, screening, exclusion, and inclusion of studies in this review. The initial database search retrieved 400 articles (237 from Scopus and 163 from Web of Science). After 173 duplicates were removed, 227 studies were screened. Of these, 212 articles were excluded for the following reasons: 54 were review articles, 5 were not in English, 91 did not focus on low‐fat products, and 62 lacked sensory analysis.

Ultimately, 15 articles met the inclusion criteria and were selected for the review. This selection process, guided by the search strategy, inclusion/exclusion criteria, and systematic screening, yielded a diverse collection of studies. These studies explore various aspects of emulsion‐based fat perception, including physicochemical changes induced by processing techniques and sensory attributes influencing consumer preferences (see Data S1). Together, they form a comprehensive basis for examining the interplay between food processing, emulsions, and fat perception within modern food science.

### 3.2 Food Application Categories

#### 3.2.1 Emulsion Use in Processed Meat Products

Among the 15 included studies, seven applied fat reduction strategies to meat products (Chappalwar et al. 2022; Diao et al. 2023; Gurinovich et al. 2020; Ozturk‐Kerimoglu et al. 2022; Pintado et al. 2021; Vargas‐Ramella et al. 2020; Yang et al. 2020). Chappalwar et al. (2022) evaluated the effect of unripened banana peel flour as a fat replacement for low‐fat chicken patties. Low‐fat chicken patties were prepared by incorporating 0%, 1%, 2% and 3% banana peel flour to replace 50% of the externally added vegetable fat in the formulation. The nutritional content, cooking yield, emulsion stability, texture, and sensory properties of the samples were compared. The emulsion containing banana peel powder presented the highest values for both the storage modulus (*G*') and loss modulus (*G*"), without a crossover point across all frequencies in the meat emulsions formulated without banana peel. In terms of physicochemical attributes, the control group presented significantly (*p* < 0.05) elevated emulsion pH, emulsion stability, product pH, water activity, and fat and cholesterol contents. Conversely, the cooking yield, moisture content, ash content, and both fat and moisture retention values markedly increased (*p* < 0.05) in the treated patients. Significant (*p* < 0.05) variations were also observed in the mineral content (except for manganese), textural, and color measurements. The sensory evaluation revealed a notable decrease (*p* < 0.05) in the scores as the concentration of the banana peel flour increased. Specifically, the sensory scores of patients treated with 3% banana peel powder were significantly (*p* < 0.05) lower than those of patients in the other treatment groups, whereas no discernible difference was detected between chicken patties treated with 1% and 2% banana peel powder. Consequently, a formulation incorporating 2.0% banana peel flour as a substitute for 50% vegetable fat to produce low‐fat chicken patties was determined to be the optimal treatment.

Diao et al. (2023) investigated the effects of lard‐based diacylglycerols (DAGs) on emulsion‐type sausages. Six types of emulsion sausages were manufactured by adding different kinds of fat: Three high‐fat treatments (500 g/kg meat) prepared with lard, glycerolized lard (GL), and purified glycerolized lard (PGL; used as control groups) and three low‐fat treatments (200 g/kg meat) prepared with lard, GL, and PGL. The samples were compared in terms of their cooking loss, texture, and sensory properties. Compared with high‐fat sausages (500 g/kg), low‐fat sausages (200 g/kg) presented greater lightness (L*), lower cooking loss, and more restricted water mobility (evidenced by slower *T*_23_ relaxation times and greater *A*_23_ peak areas), suggesting better water retention and texture properties. Compared with lard, GL and PGL had superior textural properties (hardness, springiness, cohesiveness, chewiness, and resilience), indicating that DAGs can enhance the stability and texture of low‐fat emulsions. Sensory analysis revealed that all the sensory attributes of the high‐fat samples were rated lower than those of the low‐fat samples. At the same fat content, sausages with GL and PGL were preferred over lard‐based sausages, although there were no significant differences in overall acceptability. Microstructural analysis revealed that the low‐fat samples presented a denser and more homogeneous network than did the high‐fat samples, which presented many voids. The compact structure of sausages with PGL suggested that increased DAG concentrations led to smaller and more uniform lipid droplets, enhancing the textural quality of the products.

Gurinovich et al. (2020) assessed the use of cedar oil cake as a fat replacer in semismoked sausages. In the experimental formulation, 15% of the semifat pork was replaced with cedar oil cake (a source of highly unsaturated fatty acids and high‐grade protein), and 30% of the sodium chloride was replaced with magnesium chloride. Nutritional content, water activity on days 0 and 15, and sensory analysis were carried out for comparison among the samples. Replacing 15% of semifat pork with cedar oil cake resulted in a 19.8% decrease in saturated fatty acids (SFAs), whereas monounsaturated fatty acids (MUFAs) and polyunsaturated fatty acids (PUFAs) increased by 10.2% and 24.9%, respectively. This modification improved the atherogenicity and thrombogenicity indices of the sausages, and the utility coefficient of amino acids increased from 0.83 to 0.87. Sensory evaluation, including appearance, inner color, smell, taste, and texture, highlighted the enhanced consumer appeal of sausages with cedar oil cake, underscoring its potential to enrich meat products with biologically active plant components while enhancing nutritional and sensory qualities.

Ozturk‐Kerimoglu et al. (2022) investigated the effects of incorporating microparticulate whey protein (MWP) at varying levels as a fat replacer in emulsified beef sausages. To evaluate the impacts of direct fat reduction as well as the inclusion of the MWP in reduced‐fat formulations, four different formulations were prepared. The sum of beef fat, water, and/or the MWP was fixed to 40% of the meat weight in all the treatments: (1) standard‐fat (SF) control treatment with 20% fat and 20% water; (2) reduced‐fat (RF) control treatment, formulated by reducing fat by half (10%) and adding 30% water; (3) MWP1 treatment with 10% fat, 5% MWP and 25% water; and (4) MWP2 treatment with 10% fat, 10% MWP and 20% water. Nutritional content, cooking yield, emulsion stability, texture and sensory analyses were carried out to compare the samples. The moisture content was significantly lower in the SF samples, whereas the MWP2 sample had the highest protein content, indicating effective moisture and protein retention in the MWP‐enhanced sausages. The fat content decreased progressively from the SF to the MWP2 formulations, with the RF, MWP1, and MWP2 formulations showing significant reductions, highlighting the role of the MWP in fat reduction without compromising moisture. Sensory evaluation revealed no significant differences in color, flavor intensity, or overall impression scores among sausage formulations, suggesting that fat reduction and MWP addition did not adversely affect sensory qualities. Textural analysis revealed that RF sausages had the highest hardness, potentially due to denser structures, whereas MWP formulations maintained cohesiveness similar to that of full‐fat controls, indicating that the MWP can effectively mimic the textural properties of higher fat sausages.

Pintado et al. (2021), examined the use of emulsion gels containing polyphenol extracts as animal fat replacers in frankfurters. Three types of emulsion gels were prepared: A reference gel was prepared with water, extra virgin olive oil, soy protein isolate, and a gelling agent (alginate); two further emulsion gels also included either 2.32% grape seed extract (EPG) or 1.95% extract from grape seeds and olives (EPGO). Both EPG and EPGO were designed with similar phenolic compound concentrations to ensure a high content in the frankfurter. Five different frankfurters were prepared, two of which were used as reference sausages: One with normal pork fat content (23%, N‐F) and the other with reduced pork fat content (12%, R‐F). Three different reduced‐fat (12%) frankfurters were prepared by completely replacing the pork fat with the same proportion of the corresponding emulsion gel (reference, EPG, and EPGO). Nutritional content, instrumental texture, and sensory properties were compared among the samples. The incorporation of EGs significantly reduced the fat content in frankfurters, with reduced‐fat samples showing a fat content of approximately 12%, nearly half that of the normal‐fat samples, which was approximately 24%. Recent advancements in plant‐based double emulsions, such as those integrating vegetable oils, have demonstrated their effectiveness in achieving low‐fat formulations while enhancing both sensory appeal and nutritional quality (Huang et al. 2024). This reduction aligns with the EU Regulation (EC) No 1924/2006 for “reduced fat” labelling. The reduction in saturated fatty acids (SFAs) to approximately 18.84% and the increase in monounsaturated fatty acids (MUFAs) and polyunsaturated fatty acids (PUFAs) were notable, enhancing nutritional quality. Specifically, the PUFA/SFA ratio improved, indicating a healthier lipid profile in the low‐fat frankfurters. The processing loss values ranged from 11.0% to 13.7%, with reduced‐fat frankfurters showing the highest values. The use of EGs in frankfurters resulted in comparable processing losses to those of traditional animal fats. The texture of the frankfurters was significantly influenced by the formulation, with measurements of hardness, cohesiveness, springiness, and chewiness indicating that the EGs could effectively mimic the textural properties of their full‐fat counterparts. The addition of phenolic compounds through the EGs did not adversely affect the microbiological quality of the frankfurters, maintaining their safety or extending their shelf life. The sensory evaluation conducted involved a panel that regularly consumed this type of product, indicating that the assessments were made by individuals familiar with such products' expected sensory characteristics. A rating test with fixed extremes ranging from 0 (intensely dislike) to 10 (intensely like) was employed for the sensory evaluation. Frankfurters formulated with EGs (EC, EPG, and EPGO) received similar scores for color, flavor, juiciness, texture, and general acceptability.

Vargas‐Ramella et al. (2020) explored the use of healthy oil‐in‐water gelled emulsions as partial animal fat replacers in dry‐fermented deer sausages. Four different formulations were manufactured. The reference contained 18.2% animal fat. In the other samples, 50% of the animal fat was substituted with olive, canola, or soy oil emulsions immobilized in Prosella gel. Nutritional content, instrumental color, and texture analysis were used to compare these properties among the samples. Reformulated sausages had significantly greater amounts of volatile organic compounds (VOCs), ranging from 17,854 to 19,027 AU × 10^4^/g, than did the control sausages (12,378 AU × 10^4^/g), suggesting a correlation with moisture content. Sausages formulated with vegetable oil emulsions presented different moisture levels, with reformulated batches having lower moisture contents (27.69%–29.90%) than the control (39.79%). The SFAs showed significant variations, ranging from approximately 12.88 ± 0.42 to 17.54 ± 0.65, and the PUFAs also demonstrated considerable differences. The descriptive sensory analysis indicated that meat color, odor, chewiness, and rancid flavor were significantly influenced by reformulation. Notably, compared with the control, the reformulated sausages scored higher for odor, primarily in samples with canola and soy oil. Although black pepper flavor was more pronounced in the reformulated samples, this difference was not statistically significant. A consumer acceptance test involving 68 participants evaluated the sausages on a 7‐point hedonic scale and a structured 4‐point scale for preference. The results demonstrated varied preferences, with soy oil‐reformulated sausages receiving the highest scores, indicating strong consumer preference for these sausages over the control and other reformulated batches.

Yang et al. (2020) used *Flammulina velutipes* polysaccharide nanoparticles as a fat substitute in sausages. FPOE was prepared at a ratio of palm oil to FVPN solution of 3:7. Given the inability of the Pickering emulsion (phase separation) and the possible effect on the properties of the sausages, two forms of emulsion, with or without creaming, were added to the sausages to separately replace their original fat by 5%, 10%, 15%, 20%, 25%, 30%, or 37%. Nutritional content, instrumental texture, and sensory properties were compared among the samples. Reformulated sausages presented an increase in moisture content from 53.24% to 64.85% and protein content from 11.97% to 12.76%, whereas the fat content decreased from 27.28% to 18.76%. There was a significant reduction in cooking loss, from 18.87% to 8.63%, with increasing FPOE amount in the sausages, indicating improved water retention. The emulsion improved the springiness and cohesiveness of the sausage, with a significant reduction in hardness and chewiness when the replacement amount was < 20%. Ten panelists were selected and trained for sensory evaluation. The panelists assessed the appearance, flavor, taste, and general acceptability of the sausages. A continuous scale ranging from 1 to 9 was utilized for the evaluation of sensory attributes. The study revealed no harmful effects on sensory characteristics, demonstrating that the emulsion, especially with no creaming, can be effectively used as a fat substitute at a level of 20% or less without adversely affecting the sensory characteristics of emulsified sausages.

#### 3.2.2 Application in Dairy Alternatives

Three studies investigated fat reduction in dairy‐type applications (Mohammed et al. 2019; Nasirpour‐Tabrizi et al. 2020; Zhou et al. 2019). The use of emulsion gels in dairy products has been highlighted as a functional strategy to improve texture and sensory properties while maintaining reduced fat levels (Xu et al. 2024). Wang et al. (2024) reviewed approaches to overcome flavor challenges in reduced‐fat dairy products, focusing on strategies that enhance flavor compound retention and improve sensory profiles in low‐fat formulations.

Nasirpour‐Tabrizi et al. (2020) focused on developing low‐fat spreadable cheese using different combinations of hydrocolloids to produce a low‐oil spreadable emulsion gel using flaxseed oil. RSM was applied to optimize the effects of different levels of hydrocolloids (locust bean gum = 0%–0.25%; κ‐carrageenan = 0%–2%; xanthan gum = 0%–3%; maltodextrin = 0%–20%) on the sensory attributes (hardness, spreadability acceptance, and overall acceptability) and instrumental hardness of each product. A second‐order polynomial model was used in all the cases. Optimization of the formulation by Design Expert software introduced formulations with high spreadability acceptance, desirable sensory properties, and acceptable instrumental hardness. The optimized formulation contained 0.09% locust bean gum, 1.95% κ‐carrageenan, 0.8% xanthan gum, and 10% maltodextrin, with a total flaxseed oil content of 19%, achieving the desired sensory properties and acceptable hardness. The study's response surface methodology revealed that spreadability acceptance and overall acceptability were closely correlated (*r* = 0.98), indicating that factors affecting spreadability also influenced overall product appeal. Hydrocolloid concentration adjustments had a significant effect on the emulsion gel properties, where locust bean gum above a certain threshold negatively affected spreadability due to textural changes, whereas κ‐carrageenan, xanthan gum, and maltodextrin contributed positively to the texture and acceptance of the product. The fatty acid analysis highlighted the health benefits of the emulsion gel, emphasizing the high content of mono‐ and polyunsaturated fatty acids, particularly ω‐3 fatty acids, making it a healthier alternative to conventional spreadable fats and butter. Rheological measurements demonstrated that the emulsion gel exhibited strong gel characteristics without transitioning from gel to sol even under high shear, indicating its stability and suitability as a spreadable product. Sensory evaluation conducted by 42 trained assessors via a 9‐point hedonic scale and instrumental texture analysis confirmed the formulation's high spreadability acceptance and overall sensory appeal, with specific sensory scores ranging from 1 (low acceptance) to 7.5 (high acceptance) and instrumental hardness values aimed at mimicking traditional spreads.

Zhou et al. (2022) investigated the effect of solid fat content (SFC) on the creamy mouthfeel of acid milk gels. Five kinds of blended milk fats with SFCs of 10%, 20%, 40%, 60%, and 85% were prepared. Then, acid milk gels differing in SFC were prepared. The creamy mouthfeel evaluation of acid milk gels was performed via both qualitative descriptive analysis (QDA) and temporal dominance of sensation (TDS) analysis. Five blended milk fats with SFC values ranging from 10.61% to 85.87% were prepared and used to produce acid milk gels. A greater SFC in fat droplets led to a greater degree of droplet coalescence during simulated oral processing, with the order of degree of coalescence being EG40 > EG20 > EG60 > EG10 ≈ EG85. The friction coefficients measured by the tribological methods were negatively correlated with the coalescence result, indicating that intermediate SFC (approximately 40%) promoted optimal lubrication and creamy mouthfeel. Sensory evaluation through quantitative descriptive analysis (QDA) and temporal dominance of sensation (TDS) analysis revealed that SFC significantly affected the ratings of melting, mouth coating, smoothness, and overall creaminess of acid milk gels. A medium SFC level (*~*40%) provided the best balance between a creamy mouthfeel, the longest duration of melting and smoothness, and nutritional content.

Mohammed et al. (2019) investigated functional nondairy creamer (NDC) formulations using *Nigella sativa* oil and microencapsulation techniques to improve the physical and functional properties of powdered creamers. The emulsion was processed with 40% total solids, and the drying process was performed via a spray drying technique at an inlet air temperature of 160°C to obtain the microencapsulated oil. RSM was applied to optimize the parameters of fluidized bed drying: Fluidizing time (20°C–60°C), fluid air temperature (20–50 min), and feed flow rate (1–2.5 mL/min). The measured variables were the moisture content (Y1), solubility (Y2), antioxidant activity (Y3), and total phenolic content (TPC) (Y4) of 20 simplified experimental sets. The central composite design was applied, with three coded levels for the independent variables, as well as the central, cubic, and axial points. The polynomial regression model equation was utilized, and the performance of the response surface was examined. The optimum NDC was then compared (after mixing the creamer with coffee) in terms of pH, instrumental color, viscosity, and sensory properties with those of a commercial NDC, a commercial dairy creamer, and coffee. The ideal conditions included an inlet air temperature of 50°C, a drying time of 25 min, and a feed flow rate of 1 mL/min for producing NDC with an optimal moisture content, water solubility, antioxidant activity, and thymoquinone content. Under these optimized conditions, the NDC exhibited a low moisture content (ranging from 1.43% to 7.64% on a dry basis) and high water solubility (84.23%–98.03%), indicating that successful microencapsulation enhances the creamer's stability and solubility. The study revealed significant effects of fluidizing time and temperature on the antioxidant capacity of NDC, with fluidizing time and temperature negatively affecting the antioxidant capacity and phenolic content but positively influencing the moisture content and solubility. A sensory evaluation conducted with 50 untrained panelists via a 9‐point hedonic scale for taste, aroma, color, and overall acceptability revealed high consumer acceptability for the developed NDC compared with commercial creamers, despite slight differences in specific sensory attributes. Consumer acceptance of low‐fat products is often linked to innovative sensory evaluation methods and fat replacer designs, which can enhance both sensory appeal and overall product quality (Gao et al. 2024).

#### 3.2.3 Emulsions in Salad Dressings and Sauces

Three studies applied fat reduction strategies in salad dressings (Liu et al. 2018; Mooliani and Nouri 2021, and Agyei‐Amponsah et al. 2019). Liu et al. (2018) focused on creating a microparticulate egg white protein (MEWP) as a fat mimetic in salad dressings. In single‐factor experiments, the effects of various factors (heating time, pH, protein addition, shear time, and rotational speed) on the textural properties of MEWP (firmness, consistency, cohesiveness, and index of viscosity) were studied with the other factors held constant. A uniform design was then applied to determine the optimal process parameters for MEWP production, and the rheological, textural, and sensory properties of the optimum WEWP‐salad dressing were compared with those of commercial salad dressing (CSD). A regression analysis fits the mathematical model to the experimental data, with the variance analysis showing significant impacts of the variables on the textural properties (firmness, consistency, cohesiveness, and index of viscosity) of the MEWP, all of which are significant at *p* < 0.01. The optimal operation parameters were a heating time of 13 min, a solution pH value of 3.6, an MEWP addition amount of 90 g/L, and a shear time of 60 s at a rotational speed of 10,000 rpm. Under these optimal conditions, the textural properties of MEWP produced were comparable to those of CSD, with the firmness, consistency, cohesiveness, and viscosity indices showing minimal differences between MEWP and CSD, indicating MEWP's potential as an effective fat replacer. Sensory evaluation revealed that the MEWP closely matched the appearance and texture of the CSD but had a slight egg smell, which slightly reduced its sensory score compared with that of the CSD.

Mooliani and Nouri (2021), examined the optimization of salad dressing formulations using avocado and whey protein (WP) combined with mint extract (ME). Response surface methodology (RSM) was applied to assess the impact of avocado (40%, 60%, 80%), whey protein (3%, 6.5%, 10%), and mint extract (1%, 1.5%, 2%) on the total soluble solids, viscosity, peroxide level, physical stability, and microbial and sensory attributes of processed food. A Box–Behnken design was performed on the basis of the three independent variables, and a second‐order polynomial model was utilized. The coefficient of determination (*R* ^2^), adjusted *R* ^2^ (*R* ^2^ adj), and predicted *R* ^2^ (*R* ^2^ pred) were used to estimate the quality of the model. Optimization led to an ideal formulation comprising 63.91% avocado, 7.87% WP, and 1.27% ME, which significantly enhanced the total soluble solids, viscosity, and stability of the salad dressing. This optimized salad dressing exhibited notable improvements in oxidative stability, with peroxide values (PVs) minimized to 1.04 mEq/kg oil, indicating enhanced preservation against lipid oxidation. Microbial analysis revealed a significant reduction in *Escherichia coli* counts, enhancing the microbial safety of the product. Fatty acid analysis highlighted the nutritional enhancement of the dressing, with oleic acid (omega‐9) accounting for 55.20% of the total fatty acids, followed by linoleic (omega‐6) and palmitic acids, aligning the product's fatty acid profile closer to that of olive oil and emphasizing its health benefits. The study's statistical analysis demonstrated a high degree of model fit for the optimization parameters, with significant effects (*p* < 0.05) observed for the interactions between avocado, WPC, and ME on the physicochemical properties of the salad dressing. The sensory evaluation scores ranged from 3.00 to 5.00 across the different attributes, indicating favorable organoleptic properties of the optimized salad dressing.

Agyei‐Amponsah et al. (2019) investigated starch–lipid complexes as fat replacers in mayonnaise‐type emulsions. The following four samples were compared in terms of their sensory, tribological, rheological, and textural properties: Maize starch modified by the incorporation of 1.5% (w/w) stearic acid; maize starch modified by the incorporation of 2% (w/w) monoglyceride; and commercial citrus base fat replacer and sunflower oil (standard). This study demonstrated that starch–lipid complexes significantly alter the sensorial, tribological, rheological, and textural properties of food emulsions. Specifically, compared with those of commercial fat replacer and sunflower oil, modified maize starches (with 1.5% stearic acid and 2% monoglyceride) presented lower flow behavior indices and consistency coefficients, indicating their potential as effective fat replacers with fat‐like properties, including being glossy, creamy, and smooth. The study also highlighted that these complexes were less firm, less bitter, and provided better lubricating properties than did the commercial fat replacer, making them suitable for applications such as low‐fat mayonnaise.

#### 3.2.4 Integration Into Bakery Products

Two studies investigated reducing fat content in bakery products such as sweet bread (Plazzotta, Nicoli, and Manzocco 2023) and shortbread biscuits (Schmid et al. 2020). Plazzotta, Nicoli, and Manzocco (2023) transformed upcycled soy processing waste into structured emulsions for sweet bread. Solid emulsions were prepared by emulsifying 1000, 900, 700, 600, 500, and 480 g/kg okara with 0, 100, 300, 400, 500, and 520 g/kg sunflower oil, respectively. The formulation of sweet bread containing palm margarine was modified by substituting margarine with an okara solid emulsion. The control bread samples were prepared with palm margarine (PM) or sunflower oil (SO) alone. Samples containing okara were obtained by adding okara and SO to the formulation either as separate ingredients (O + SO) or as an emulsion (500 g oil/kg, OE). Substitution was performed while maintaining a constant overall dough water content, thus taking into consideration the moisture content of PM (190 g/kg) and okara (760 g/kg) and the ratio among the other ingredients. The nutritional content and rheological properties of the different emulsions were analyzed, together with the nutritional content, specific volume, instrumental color, firmness, and sensory properties of the sweet bread. The preparation of stable structured emulsions with up to 520 g of oil kg^−1^, demonstrating oil holding capacities higher than 75%, was achieved by high‐shear mixing of okara with liquid oil. Plant‐based double emulsions, such as those incorporating vegetable oils, have shown great potential in creating low‐fat formulations with improved sensory and nutritional profiles (Huang et al. 2024). Microstructural analyses revealed an even distribution of the oil phase within the okara matrix, suggesting the emulsification capability of the okara fiber–protein network. The replacement of palm margarine with okara emulsions in sweet bread formulations resulted in significant reductions in saturated fatty acids (over 50%) and increases in protein and fiber contents. Comparisons of the physical and sensory properties of bread containing okara emulsions to those containing palm margarine emulsions revealed that the former had similar physical and sensory properties, except for greater oiliness and rancidity.

Schmid et al. (2020), replaced palm fat with physically modified Swiss rapeseed oil in bakery products. A total of 4 g of wax was dissolved in 96 g of rapeseed oil (MC wax crystals). Two different types of particles are used to stabilize two different types of emulsions: Microcrystalline cellulose to stabilize O/W emulsions (MCC emulsions) and rapeseed press cake to stabilize W/O emulsions (RPC emulsions). A total of 3 g of microcrystalline cellulose, 5 g of powdered milk protein concentrate, and 1 g of guar kernel flour were dissolved in 91 g of tap water at 40°C and cooled to 20°C (MCC foam). Five different shortbread biscuits were formulated using palm fat, rapeseed oil, MCC emulsions, RPC emulsions, MC wax crystals, or MCC foam as the fat fraction. Instrumental color, texture, rheological and sensory properties were compared among the samples. This study revealed that physically modified samples had significantly greater viscosities than did pure rapeseed oil, with viscosities ranging between 1.05 and 23.02 Pas, whereas the viscosity of palm fat was 393.70 Pas. The viscosity of emulsified samples containing press cake increased by 19% from day 0 to day 5, whereas the viscosity of samples with microcrystalline (MC) wax crystals decreased by 42%. Microstructural assessment via light microscopy revealed that the enrichment of rapeseed oil with microcrystalline wax resulted in the formation of evenly distributed crystals in the sample, closely resembling the microstructure of palm fat, albeit with smaller and more densely packed palm fat crystals.

### 3.3 Methodological Flaws in Sensory Analysis

Table 1 (and Data S2) synthesizes the sensory and physicochemical analyses conducted in the included studies. Variability in methodologies, sensory panel training, and the range of sensory attributes assessed were common issues.

**TABLE 1:** Synthesis of the physicochemical and sensory analyses carried out in the included studies.

Study	Physico–chemical analysis	Nutritional content	Color	Process yield	Texture analysis	Tribology	Rheology	Sensory analysis
Agyei‐Amponsah et al. (2019)					X	X	X	9 T
Chappalwar et al. (2022)		X						7 T
Diao et al. (2023)				X	X			10 U
Gurinovich et al. (2020)	X	X						n/a U
Liu et al. (2018)					X		X	10 U
Mohammed et al. (2019)	X		X		X			50 U
Mooliani and Nouri (2021)	X				X			20 U
Nasirpour‐Tabrizi et al. (2020)					X			14 T
Ozturk‐Kerimoglu et al. (2022)	X	X		X				10 T
Pintado et al. (2021)		X		X	X			n/a U
Plazzotta, Nicoli, and Manzocco (2023)	X	X	X		X		X	20 C
Schmid et al. (2020)			X		X			14 U
Vargas‐Ramella et al. (2020)		X	X		X			15 T, 68 C
Yang et al. (2020)		X		X	X			10 T
Zhou et al. (2022)	X						X	10 T

Abbreviations: C, consumers; n/a, not available; T, trained; U, untrained.

Many of the reviewed studies did not strictly adhere to established standards, such as ISO 8586:2012 for the selection, training, and monitoring of assessors and ISO 11132:2021 for the performance and analytical capacity of the panel members. These standards are critical for ensuring the reliability and validity of sensory evaluations (Talens et al. 2024).

Several studies did not specify whether they used trained or untrained panels, which is a significant omission. The training of assessors is essential for ensuring the consistency and reliability of sensory evaluations. For example, Diao et al. (2023) did not specify the training level of their panel, which could impact the consistency of their results. Similarly, Gurinovich et al. (2020) did not detail the training process of their panelists, raising questions about the reliability of their sensory data.

In terms of the sensory attributes evaluated, there was considerable variation among studies. Some studies, such as those by Chappalwar et al. (2022) and Vargas‐Ramella et al. (2020), have assessed a comprehensive range of sensory attributes, including appearance, flavor, texture, and overall acceptability. In contrast, other studies, such as Mooliani and Nouri (2021), have only assessed overall acceptability, limiting the depth of sensory insight that can be gleaned from their findings.

The number of sensory attributes evaluated also varied widely Chappalwar et al. (2022) evaluated eight attributes, making it one of the most comprehensive sensory evaluations in the review. Conversely, studies such as those of Mooliani and Nouri (2021) and Liu et al. (2018) have evaluated far fewer attributes, which may limit their ability to capture the full sensory profile of the products.

Another significant gap is the lack of standardization in sensory evaluation methods. Some studies have used subjective measures without adequate calibration or validation against standard protocols. This lack of standardization can lead to variability and reduce the comparability of results across different studies.

Additionally, some studies failed to report key details such as the number of assessors, the presentation order of the samples, and the statistical methods used to analyze the data. These omissions can introduce biases and affect the robustness of the sensory evaluation. For example, Nasirpour‐Tabrizi et al. (2020) conducted a detailed statistical analysis but did not provide sufficient information on the randomization of sample presentation. This could lead to order effects, where the sequence of sample presentation influences the sensory ratings.

Moreover, the environmental conditions used during sensory evaluations, such as lighting, temperature, and the use of sensory booths, have not been consistently reported. These factors can significantly influence sensory perceptions and should be standardized to ensure reliable and valid results.

### 3.4 Proposed Strategies for Sensory Analysis in Low‐Fat Emulsion Research

To address the identified methodological flaws and improve the quality of sensory analysis in low‐fat emulsion research, the following strategies are proposed:

1. Researchers should adhere to established standards for sensory analysis, such as ISO 8586:2012 for the selection, training, and monitoring of assessors and ISO 11132:2021 for the performance and analytical capacity of sensory panels. This ensures that the sensory evaluations are reliable and reproducible.
2. The training of assessors should be thorough and well documented. This includes training in the specific attributes to be evaluated, the use of scales, and the avoidance of bias. Regular calibration sessions should be conducted to maintain the panel's performance.
3. Studies should provide detailed reporting of the sensory analysis methodologies, including the number of assessors, their training level, the presentation order of samples, and the statistical methods used. This transparency allows for better reproducibility and comparability of results.
4. Sensory evaluation methods should be standardized across studies. This includes the use of validated scales and protocols for assessing sensory attributes. Standardization reduces variability and enhances the reliability of sensory data.
5. Where feasible, studies should consider the use of a combination of trained and untrained panelists to capture both expert and consumer perspectives. This approach provides a comprehensive understanding of the sensory attributes and consumer acceptability of low‐fat emulsions.
6. The environmental conditions during sensory evaluations should be controlled and reported. This includes consistent lighting, temperature, and the use of sensory booths to minimize external influences on sensory perceptions.
7. Studies should aim to evaluate a comprehensive range of sensory attributes, including appearance, flavor, texture, and overall acceptability. A detailed sensory profile provides a more complete understanding of a product's sensory characteristics.
8. The order of sample presentation should be randomized, and the sensory evaluations should be replicated to ensure the robustness of the results. This helps mitigate order effects and enhances the reliability of the data.
9. Researchers should obtain ethical approval for sensory studies involving human participants and report this in their publications. This ensures that the studies adhere to ethical standards and that the rights of the participants are protected.

The implementation of these strategies allows researchers to enhance the quality and reliability of sensory analysis in low‐fat emulsion research, ultimately leading to a better understanding and development of consumer‐acceptable low‐fat food products.

## 4 Conclusions

The evidence presented in this review highlights the significant role of formulation and processing techniques in the development of low‐fat emulsions for various food applications. The physicochemical properties and sensory attributes of these products are critical to their acceptance and success on the market.

In meat products, the use of fat replacers such as microparticulated whey protein and lard‐based diacylglycerols has shown promising results in improving the textural properties and overall acceptability of low‐fat sausages. Sensory evaluations by trained panels and consumers indicate that these innovations can maintain the desirable attributes of traditional high‐fat products.

Dairy‐type products, including low‐fat spreadable cheeses and emulsified milk gels, have benefited from the incorporation of hydrocolloids and other stabilizing agents. These additions increase the stability and sensory characteristics of the products, making them suitable alternatives to their full‐fat counterparts. Salad dressings, including mayonnaise, have also been successfully reformulated with fat mimetics such as microencapsulated egg white proteins and avocado‐based emulsions. These formulations not only reduce fat content but also improve the spreadability and sensory appeal of the dressings, as evidenced by both instrumental and sensory analyses. In bakery products, the replacement of palm fat with alternatives such as physically modified Swiss rapeseed oil has demonstrated the potential to produce healthier baked goods without compromising texture or flavor. The use of structured emulsions and fat replacers in sweet bread formulations has led to products with reduced saturated fatty acids and enhanced nutritional profiles.

In conclusion, while some studies have provided comprehensive and methodologically sound sensory evaluations, many have significant methodological flaws and gaps. These include insufficient training of assessors, variability in the number and type of sensory attributes evaluated, lack of standardization in methods, and incomplete reporting of key details. Addressing these gaps is crucial for improving the reliability and validity of sensory analysis in low‐fat emulsion research. Future studies should adhere to established sensory analysis standards, ensure thorough training of assessors, and provide complete and transparent reporting of their methodologies.

## Author Contributions

**Clara Talens:** conceptualization (equal), data curation (equal), formal analysis (equal), funding acquisition (equal), methodology (equal), writing – original draft (lead). **Saioa Alvarez‐Sabatel:** conceptualization (equal), writing – review and editing (supporting). **Esther Sanmartín:** conceptualization (equal), writing – review and editing (supporting). **Laura Garcia‐Fontanals:** data curation (equal), formal analysis (equal). **Pau Talens:** writing – review and editing (lead).

## Conflicts of Interest

The authors declare no conflicts of interest.

## Supporting information


Data S1.

Data S2.

## Data Availability

The data supporting this article have been included as part of the Supporting Information. (Data S1) Characteristics of the included studies. (Data S2) An organized spreadsheet that serves as a repository of data from the included articles and is divided into two sheets. The first sheet details the physicochemical and sensory properties of the products developed. The second sheet describes the sensory attributes included in each study per food category.
