# Supplementary material for: Comprehensive Sensory Evaluation in Low‐Fat Emulsions: A Systematic Review of Diverse Food Applications
Source: Food Sci Nutr. 2024 Dec 20;13(1):e4700. doi: 10.1002/fsn3.4700 (PMC11717037; doi:10.1002/fsn3.4700)
Supplement: Supplementary file 1 — Data S1. [file FSN3-13-e4700-s002.docx]

Supplementary material S1: Characteristics of the included studies

| Author, Year | Emulsion type | Aim of the study | Methodology | Ingredients used | Food application |
| --- | --- | --- | --- | --- | --- |
| Agyei-Amponsah et al., 2019 | Simple Emulsions | Investigate starch–lipid complexes as fat replacers | Sample 1: modified maize starches by incorporating stearic acid at 1.5% (w/w). Sample 2: modified maize starches by incorporating monoglyceride at 2% (w/w). Control: Commercial citrus-base fat replacer | Starch, lipid complexes: stearic acid, monoglyceride and maize starch. | Mayonnaise-type emulsions |
| Chappalwar et al., 2022 | Simple Emulsions | Evaluate the effect of unripened banana peel flour as fat replacer of ultra-low fat chicken patties. | Low fat chicken patties (> 5%) were prepared with incorporation of banana peel flour at 0% (C), 1% (BP1), 2% (BP2) and 3% (BP3) levels separately to replace 50% externally added vegetable fat in formulation and evaluated for various quality characteristics and sensory attributes. | Banana peel flour | Chicken Patties |
| Diao et al., 2023 | Simple Emulsions | Evaluate the effects of lard-based diacylglycerols on emulsion-type sausages | Six types of emulsion sausages were manufactured by adding different kinds of fat: three high-fat treatments (500 g/kg meat) prepared with lard, GL, and PGL (used as control groups); and three low-fat treatments (200 g/kg meat) prepared with lard, GL, and PGL, respectively. | Lard-based diacylglycerols | Emulsion-type sausages |
| Gurinovich et al., 2020 | Simple Emulsions | Develop semi-smoked sausages with cedar oil cake | 15% of the semi-fat pork (50% fat) was replaced by cedar oil cake (source of highly unsaturated fatty acids and high-grade protein), and 30% of sodium chloride – by magnesium chloride. | Cedar oil cake | Semi-smoked sausages |
| Liu et al., 2018 | Simple Emulsions | Reduce fat content in salad dressings using microparticulated egg white proteins (MEWP) | In single factor experiments, the effect of changing levels of a factor (heat time, pH value, protein addition, shear time and rotational speed) on the textural properties of MEWP (firmness, consistency, cohesiveness, and index of viscosity) was studied with the other factors being held constant. Uniform design was then applied to determine the optimal process parameters of MEWP production and compared to commercial salad dressing (34.9% fat) | Microparticulated egg white proteins | Salad dressings |
| Mohammed et al., 2019 | Emulsion gel | Produce a low-fat, non-dairy creamer using Nigella sativa oil | Response Surface Methodology (RSM) was applied to optimize the parameters of fluidized bed drying: fluidizing time (20–60 °C); fluid air temperature (20–50 min); and feed flow rate (1–2.5 mL/min). The measured variables were moisture content, solubility, antioxidant activity using DPPH-IC50, and total phenolic content (TPC) of twenty simplified experimental sets. The central composite design was applied, with three coded levels for the independent variables, as well as the central, cubic, and axial points. The polynomial regression model equation was utilized, and the performance of the response surface was examined. | *Nigella sativa* oil | Non-dairy creamer |
| Mooliani et al., 2021 | Simple Emulsions | Optimize salad dressing emulsions based on avocado and whey protein | RSM was applied to assess the impact of avocado (40, 60, 80%), whey protein (3, 6.5, 10%), and mint extract (1, 1.5, 2%) on total soluble solids, viscosity, peroxide level, physical stability, and microbial and sensory attributes of processed food. A Box Behnken design (BBD) was performed based on the three independent variables and a second-order polynomial model was utilized. The coefficient of determination (R2), adjusted-R2 (R2 adj), and predicted-R2 (R2 pred) are used to estimate the quality of the model. | Avocado, whey protein, mint extract | Salad dressing emulsions |
| Nasirpour-Tabrizi et al., 2020 | Emulsion Gels | Create a healthy alternative for spreadable fats using flaxseed oil | RSM was applied to optimize the effect of different levels of hydrocolloids (LBG = 0%–0.25%; **CAR** = 0%–2%; XAN = 0%–3%; MD = 0%–20%) on the sensory attributes (hardness, spreadability acceptance and overall acceptability) and instrumental hardness of each product. A second-order polynomial model was used. | Flaxseed oil, locust bean gum (LBG), k-carrageenan (CAR), xanthan gum (XAN) and maltodextrin (MD) | Spreadable cheese |
| Ozturk-Kerimoglu et al., 2022 | Simple Emulsions | Utilize microparticulated whey protein (MWP) as fat replacers in emulsified beef sausage | For evaluation of the impacts of direct fat reduction as well as the inclusion of MWP to reduced-fat formulations, four different formulations were prepared. The sum of beef fat, water and/or MWP was fixed to 40% of the meat weight in all treatments: (i) standard-fat control treatment (SF) with 20% fat and 20% water; (ii) reduced-fat control treatment (RF), formulated by reducing fat by half (10%) and adding 30% water; (iii) MWP1 treatment with 10% fat, 5% MWP and 25% water; and (iv) MWP2 treatment with 10% fat, 10% MWP and 20% water. | Microparticulated whey protein | Emulsified beef sausage |
| Pintado et al., 2021 | Emulsion Gels | Use emulsion gels containing polyphenol extracts as animal fat replacers in frankfurters | Three types of emulsion gels were prepared: the reference was prepared with water, extra virgin olive oil, soy protein isolate and a gelling agent (EC); two further EGs also included either 2.32% of grape seed extract (EPG) or 1.95% of extract from grape seed and olive (EPGO). Both EPG and EPGO were designed with similar phenolic compounds concentration to ensure a high content in the frankfurter.  Five different frankfurters were prepared, two of these as reference sausages, one with normal pork fat content (23%, N-F), and the other with reduced pork fat content (12%, R-F). Three different reduced-fat (12%) frankfurters were prepared by totally replacing the pork fat with the same proportion of the corresponding EG (R-EC, R-EPG and R-EPGO). | Grape seed solid polyphenol extract and grape seed and olive solid polyphenol extract | Frankfurters |
| Plazzotta et al., 2023 | Pickering Emulsions | Upcycled soy processing waste into structured emulsions for sweet bread | Solid emulsions were prepared by emulsifying 1000, 900, 700, 600, 500 and 480 g/kg of okara with 0, 100, 300, 400, 500, and 520 g/kg of sunflower oil, respectively.  The formulation of sweet bread containing palm margarine was modified by substituting margarine with okara solid emulsion (target 20% fat). Control bread samples were prepared using palm margarine (PM) and sunflower oil (SO) solely. Samples containing okara were obtained by adding to the formulation okara and SO either as separate ingredients (O + SO) or as an emulsion (500 g oil/kg, OE). Substitution was performed while maintaining constant the overall dough water content, thus taking into consideration the moisture content of PM (190 g/kg) and okara (760 g/kg), and the ratio among the other ingredients. | Okara, liquid oil | Sweet bread |
| Schmid et al., 2020 | Pickering Emulsions | Replace palm fat with physically modified Swiss rapeseed oil in bakery products | 4 g of wax were dissolved in 96 g of rapeseed oil (MC wax crystals).  Using two different types of particles to stabilize two different types of emulsion: microcrystalline cellulose to stabilize O/W emulsion (MCC emulsion), and rapeseed press cake to stabilize W/O emulsion (RPC emulsion).  3 g of microcrystalline cellulose, 5 g of powdered milk protein concentrate, and 1 g of guar kernel flour were dissolved in 91 g tap water at 40°C and cooled to 20°C (MCC foam).  Five different shortbread biscuits were formulated using palm fat, rapeseed oil, MCC emulsion, RPC emulsion, MC wax crystalls or MCC foam as the fat fraction. | (i) Microcrystalline wax and rapeseed oil  (ii) Microcrystalline cellulose (MCC), rapeseed cake, rapeseed oil and water  (iii) MCC, water, powdered MPC, guar kernel flour, and air  (iv) Palm fat, rapeseed oil or palm fat replacer | shortbread biscuits |
| Vargas-Ramella et al., 2020 | Emulsion Gels | Improve nutritional characteristics of dry-fermented deer sausage | Four different formulations were manufactured. The reference contained 18.2% animal fat. In the other samples, 50% of animal fat was substituted by olive, canola, and soy oil emulsions immobilized in Prosella gel. | Olive oil, canola oil, soy oil and *Prosella* gel | Dry-fermented deer sausage |
| Yang et al., 2020 | Pickering Emulsions | Use *Flammulina velutipes* (FVPN) polysaccharide nanoparticles as a fat substitute | The FPOE was prepared according to the ratio of palm oil to FVPN solution being 3:7. Given the instability of Pickering emulsion (phase separation) and the possible effect on the properties of the sausages, two forms of emulsion, with or without creaming, were added to sausages to separately replace their original fat by 5%, 10%, 15%, 20%, 25%, 30% or 37%. | (i) Polysaccharide Nanoparticles from FVPN and palm oil  (ii) Pork back fat and FVPN-palm oil emulsions | Emulsified sausages |
| Zhou et al., 2022 | Emulsion Gels | Investigate creamy mouthfeel of acid milk gels affected by solid fat content | Five kinds of blended milk fats with SFC of 10, 20, 40, 60 and 85% were prepared. Then, acid milk gels differing in SFC values were prepared (E10, E20, E40, E60 and E85). The creamy mouthfeel evaluation of acid milk gels was performed using both QDA and TDS (temporal dominance of sensations) analysis. | Anhydrous milk fat, glyceryl stearate and oleic acid glyceride | Acid milk gels |
